# Gut microbiota implication in diabetic kidney disease: mechanisms and novel therapeutic strategies

**DOI:** 10.1080/0886022X.2025.2517402

**Published:** 2025-06-25

**Authors:** Yujie Zhang, Jianbo Qing, Yasin Abdi Saed, Yafeng Li

**Affiliations:** a School of Pharmaceutical Science, Sun Yat-Sen University, Guangzhou, China; b Department of Nephrology, Sir Run Run Shaw Hospital, Zhejiang University School of Medicine, Hangzhou, China; c Department of Nephrology, Shanxi Provincial People’s Hospital (Fifth Hospital) of Shanxi Medical University, Taiyuan, China; d Department of Nephrology, Eighth Affiliated Hospital of Sun Yat-sen University, Shenzhen, China

**Keywords:** Diabetic kidney disease, gut microbiota, probiotics, prebiotics, synbiotics, fecal microbiota transplantation

## Abstract

Diabetic kidney disease (DKD) is one of the leading causes of chronic kidney disease and end-stage renal disease worldwide, predominantly driven by the rise in type 2 diabetes mellitus. Recent evidence highlights the crucial role of gut microbiota dysbiosis in the development and progression of DKD. Dysbiosis, characterized by a reduction in beneficial short-chain fatty acid-producing bacteria and an increase in pathogenic species such as *Proteobacteria* and *Bacteroides*, exacerbates systemic inflammation, insulin resistance, and kidney damage through mechanisms like increased intestinal permeability and the production of pro-inflammatory metabolites like lipopolysaccharides. This review explores the impact of specific bacterial taxa on DKD risk and progression, such as *Alistipes*, *Subdoligranulum*, and their interactions with metabolic pathways. Furthermore, we discuss novel therapeutic strategies targeting gut microbiota, including probiotics, prebiotics, synbiotics, and fecal microbiota transplantation, which have shown promise in ameliorating DKD symptoms. However, the heterogeneity of gut microbiota across individuals and the challenges in treatment standardization call for personalized approaches and further research into the gut-kidney axis.

## Graphical Abstract

**Figure:**
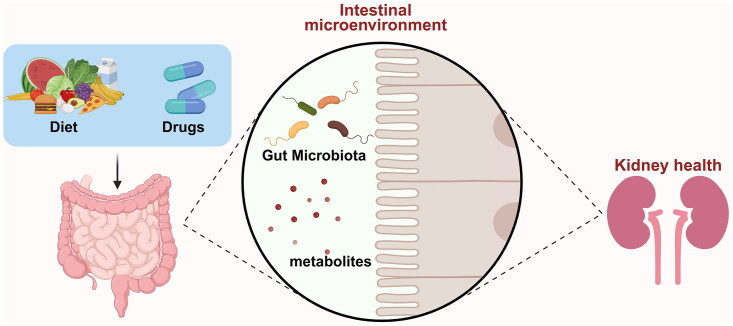

## 1. Introduction

Diabetic kidney disease (DKD) is a common chronic complication in individuals with diabetes, characterized by the progressive decline in renal function, which can potentially lead to end-stage renal disease (ESRD) [1,2]. Affecting approximately 20–40% of individuals with diabetes, DKD is a major cause of chronic kidney disease (CKD) and ESRD worldwide, accounting for nearly 50% of all ESRD cases [3,4]. The incidence of DKD has significantly increased in recent decades, driven by the global rise in type 2 diabetes mellitus (T2DM), which is closely associated with factors such as obesity, sedentary lifestyles, and aging populations [5–7]. As a result, DKD presents a substantial challenge to healthcare systems worldwide [2].

The clinical importance of DKD extends beyond renal impairment, as it is strongly linked to increased cardiovascular morbidity and mortality [8,9]. Patients with DKD are at a significantly higher risk of cardiovascular events such as myocardial infarctions and strokes, which diminishes their quality of life and escalates healthcare costs [10]. Symptoms range from early-stage proteinuria and mild renal function decline to advanced renal failure, edema, fatigue, and anemia, adversely affecting daily activities and potentially leading to psychological stress and depression [11,12]. Effective management of DKD requires long-term healthcare resources, including regular monitoring, pharmacotherapy, dialysis, and potentially kidney transplantation [13]. Early diagnosis and intervention are essential to slow DKD progression, yet many patients are diagnosed at advanced stages due to the subtle nature of early symptoms, leading to delayed treatment.

The gut microbiota, comprising a vast community of microorganisms including bacteria, viruses, fungi, and other microbes, plays essential roles in maintaining human health [14,15]. These microorganisms aid in the digestion of complex carbohydrates, producing short-chain fatty acids (SCFAs) such as butyrate, propionate, and acetate, which not only provide energy for intestinal cells but also exhibit anti-inflammatory properties [16,17]. Moreover, the gut microbiota is crucial for the development and regulation of the immune system, influencing both pro-inflammatory and anti-inflammatory responses and maintaining intestinal barrier integrity [18–21]. Dysbiosis, or an imbalance in the gut microbiota, is linked to various diseases, including obesity, insulin resistance, and type 2 diabetes, which contribute to metabolic disorders through increased intestinal permeability and systemic inflammation [22]. Additionally, gut dysbiosis has been implicated in inflammatory bowel disease and irritable bowel syndrome, exacerbating inflammation and altering gut motility [23,24]. Emerging research also highlights the role of the gut-brain axis, where dysbiosis is associated with mental health disorders and neurodegenerative diseases like Parkinson’s disease and Alzheimer’s disease [25–27].

Recent studies have emphasized the significant role of gut microbiota in the development and progression of DKD. In DKD patients, gut microbiota composition undergoes significant alterations, with a reduction in beneficial bacteria and an increase in pathogenic species, contributing to systemic inflammation and oxidative stress, which exacerbate kidney damage [28–30]. Specific bacterial taxa, such as *Alistipes* and *Subdoligranulum*, have been linked to disease severity and clinical parameters like urinary protein levels, indicating their potential as therapeutic targets [31,32]. Additionally, microbial metabolites, such as SCFAs and trimethylamine N-oxide (TMAO), play crucial roles in modulating host metabolism and immune responses, with SCFAs supporting anti-inflammatory pathways and elevated TMAO levels being associated with increased cardiovascular risks and renal fibrosis [19,33]. Targeting these microbial pathways offers promising therapeutic strategies for managing diabetic kidney disease.

To gain a deeper understanding of the specific mechanisms by which the gut microbiota affects DKD, the next section will examine the roles these microorganisms play in the progression of the disease.

## 2. Microbiota and diabetic kidney disease development

While DKD is one of the most common causes of CKD, it is important to recognize that CKD encompasses a wide range of etiologies, including hypertensive nephrosclerosis, IgA nephropathy, and membranous nephropathy [34]. These diverse etiologies differ in their pathophysiology, metabolic background, and systemic influences, and the differentiation may also extend to their gut microbiota profiles. Although direct comparisons between DKD and nondiabetic CKD in terms of gut microbiota composition are scarce, substantial evidence indicates that individuals with T2DM—the underlying condition in DKD—harbor distinct gut microbial signatures. These include reduced microbial diversity, depletion of SCFA-producing bacteria, and enrichment of pro-inflammatory taxa compared to non-diabetic individuals [35–37]. The gut microbial environment in DKD is likely shaped by a unique combination of both renal dysfunction and diabetic metabolic stress, further distinguishing it from other CKD subtypes. These differences suggest that the gut microbiota in DKD may also differ from that in nondiabetic forms of CKD, supporting the rationale for studying DKD as a distinct entity in microbiome research.

**Figure 1.**
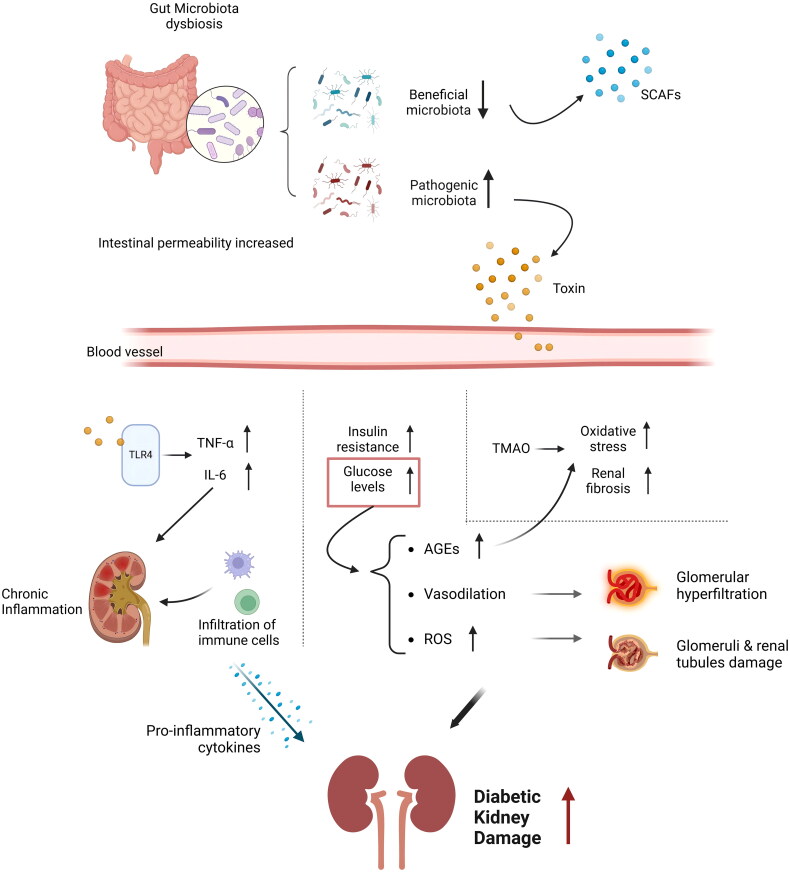
Gut microbiota’s role in diabetic kidney disease (DKD).

The pathogenesis of DKD is complex, it involves multiple metabolic and hemodynamic factors. Chronic hyperglycemia leads to a series of metabolic disturbances, including the formation of advanced glycation end-products (AGEs). These AGEs interact with their receptors, which triggers oxidative stress and inflammatory responses that result in kidney damage [38]. Additionally, hyperglycemia-induced vasodilation causes glomerular hyperfiltration, increasing the pressure and damage to the glomeruli [39]. Excessive reactive oxygen species (ROS) production in a hyperglycemic environment exacerbates oxidative stress, further damaging the glomeruli and renal tubules [40–42]. Hyperglycemia also activates the polyol pathway, protein kinase C pathway, and hexosamine pathway, collectively contributing to glomerulosclerosis and mesangial expansion[43]. Moreover, in a diabetic environment, the levels of pro-inflammatory cytokines such as TNF-α, IL-6, and TGF-β are elevated, which exacerbate kidney inflammation [22] and fibrosis through multiple pathways [42,44]. Chronic inflammation also leads to the infiltration of immune cells like macrophages and T cells into renal tissues, which then further release pro-inflammatory cytokines and worsening kidney damage [45] (Figure 1).

The gut-kidney axis represents a complex and dynamic bidirectional communication network linking the gastrointestinal tract and the kidneys. This intricate relationship involves a multifaceted interplay between the gut microbiota, their metabolites, and the immune system, which can significantly impact kidney health [46]. In the context of DKD, disruptions within the gut-kidney axis are increasingly recognized as key contributors to disease development and progression. Gut dysbiosis, a hallmark of DKD, is characterized by an imbalance in microbial composition, often manifesting as reduced microbial diversity, increased abundance of harmful bacteria, and depletion of beneficial species [47]. This dysbiosis can lead to a cascade of detrimental effects, including increased intestinal permeability, allowing harmful substances like uremic toxins to leak into the bloodstream and damage the kidneys [48]. Additionally, gut dysbiosis can trigger chronic inflammation and immune dysregulation, further exacerbating kidney injury [49]. Overall, gut microbiota composition influences kidney function through multiple mechanisms, including modulating immune responses, producing microbial metabolites such as SCFAs and TMAO, disrupting gut barrier integrity, and promoting systemic inflammation *via* LPS translocation. These pathways collectively contribute to the development and progression of DKD. The following sections will elaborate on these mechanisms in detail.

Recent Mendelian randomization studies have provided strong evidence for the causal relationship between gut microbiota and DKD. For example, Liu et al. explored the causal relationship between specific microbiota and diabetic complications using Mendelian randomization and found that changes in gut microbiota might be a key factor in the progression of diabetic complications, particularly in the development of DKD [50]. Additionally, Zhang et al. applied a similar method and further validated the bidirectional causal relationship between gut microbiota and DKD, suggesting that microbiota interventions may help slow the progression of the disease [51]. More recently, Dong et al. conducted a two-sample Mendelian randomization study and identified specific microbial taxa, including *Bacteroides* and *Ruminococcus*, as potentially causal in DKD pathogenesis [52]. Similarly, Lin and Chen integrated whole-genome sequencing datasets and provided further support for a causal link between gut microbial signatures and DKD risk [53]. These studies provide important causal evidence for a deeper understanding of the relationship between gut microbiota and DKD. Next, we will focus on discussing the possible mechanisms by which gut microbiota influences DKD.

### 2.1. Increased intestinal permeability and bacterial translocation

Gut dysbiosis, a state of imbalance in the gut microbial composition, plays a crucial role in the pathogenesis of DKD by compromising the integrity of the intestinal barrier and facilitating the translocation of harmful substances into the systemic circulation. Normally, the intestinal epithelium acts as a tightly regulated barrier, selectively permitting the passage of nutrients while preventing the entry of harmful bacteria and their components [54]. However, in DKD, the gut microbiota undergoes significant alterations, often characterized by a decrease in beneficial bacteria and an increase in harmful ones. These changes can disrupt the intestinal barrier by impairing tight junction proteins, leading to increased intestinal permeability, often referred to as a ‘leaky gut’ [55]. Consequently, harmful substances, including bacteria and their toxins, known as bacterial translocation, can leak from the gut lumen into the bloodstream, triggering systemic inflammation and immune responses that directly target and damage the kidneys, exacerbating DKD progression [56]. Moreover, an increased abundance of harmful bacteria like *Proteobacteria* and *Bacteroides* could produce LPS. LPS can translocate into circulation due to impaired gut barrier function, activating TLR4 on immune cells. This activation triggers the NF-κB pathway, leading to the release of pro-inflammatory cytokines (such as TNF-α, IL-6, IL-1β), which exacerbate renal inflammation and fibrosis.

### 2.2. Production of uremic toxins

Uremic toxins are a group of metabolites that accumulate in the bloodstream when the kidneys are not functioning properly and are unable to effectively filter waste products from the blood [57]. These toxins, poorly cleared by the compromised kidneys, alter the composition and function of the gut microbiota, leading to dysbiosis. This altered gut environment further contributes to the problem by increasing the production and absorption of uremic toxins, creating a vicious cycle [58]. The gut microbiota in individuals with DKD exhibit a decrease in beneficial bacteria and a surge in harmful ones, leading to increased production of uremic toxins such as indoxyl sulfate (IS) and p-cresyl sulfate (PCS) [30]. These toxins, normally excreted by healthy kidneys, accumulate in the bloodstream due to impaired kidney function in DKD. Uremic toxins play a significant role in the pathogenesis of DKD [59]. These toxins exert direct toxic effects on various renal cells, including podocytes, mesangial cells, and tubular epithelial cells, leading to cellular damage, inflammation, and fibrosis. For instance, IS, a well-known uremic toxin, has been shown to induce oxidative stress, apoptosis, and epithelial-to-mesenchymal transition in renal cells, contributing to glomerular sclerosis and interstitial fibrosis, hallmarks of DKD progression [60]. The accumulation of toxins such as IS and PCS contributes to systemic inflammation and oxidative stress by activating aryl hydrocarbon receptor and inducing NOD-, LRR- and pyrin domain-containing protein 3 inflammasome activation, further damaging kidney cells and exacerbating DKD progression [61].

### 2.3. Alteration of bile acid metabolism

Bile acids, primarily known for their role in dietary fat digestion and absorption, have recently emerged as significant contributors to the pathogenesis of DKD [62]. Primarily synthesized from cholesterol in the liver, bile acids are metabolized by the gut microbiota into a diverse pool of secondary bile acids [63]. In the context of DKD, alterations in gut microbiota composition can disrupt bile acid metabolism, leading to an accumulation of specific bile acids that exert detrimental effects on kidney health [64]. These effects include direct cytotoxicity to renal cells, activation of inflammatory pathways, and promotion of fibrosis. Furthermore, bile acids can exacerbate insulin resistance and dyslipidemia, key drivers of DKD progression [65]. Moreover, bile acids modulate immune responses by interacting with nuclear receptors such as farnesoid X receptor (FXR) and G protein-coupled bile acid receptor 1 (TGR5) [66]. Both FXR and TGR5 are known to exert renal protective effects in DKD by regulating lipid metabolism, oxidative stress, and inflammation [67]. Studies have demonstrated that FXR and TGR5 activation can inhibit NF-κB signaling, reduce the release of pro-inflammatory cytokines (TNF-α, IL-6, IL-1β), and suppress macrophage infiltration, thereby mitigating kidney inflammation and fibrosis [68]. Conversely, FXR and TGR5 downregulation in DKD is associated with increased inflammation and disease progression.

### 2.4. SCFAs deficiency

SCFAs, primarily acetate, propionate, and butyrate, are produced by the fermentation of dietary fiber by gut microbiota [17]. These metabolites play a crucial role in maintaining gut and kidney health, forming a crucial link’ in the gut-kidney axis, which is particularly relevant in diabetic disease. SCFAs activate G-protein-coupled receptors (GPR41 and GPR43), enhancing intestinal barrier integrity and exerting anti-inflammatory effects by inhibiting NF-κB signaling, suppressing the release of pro-inflammatory cytokines, and promoting the production of anti-inflammatory cytokines, thus mitigating inflammation in both the gut and kidneys [69]. SCFAs contribute to intestinal barrier integrity, reducing leakage of pro-inflammatory bacterial products into circulation, which can be detrimental to kidney function [70]. Moreover, SCFAs improve insulin sensitivity and glucose homeostasis, key factors in managing diabetes and preventing DKD [71].

Table 1 summarizes the changes in gut microbiota in DKD, including SCFA-producing microbes, LPS-producing microbes, and potential probiotics and pathogenic bacteria. These microbial changes are closely associated with the risk and progression of DKD. This table focuses on bacterial alterations observed in DKD-related gut microbiota. While the gut microbiota also includes the virome and mycobiome, research on their specific roles in DKD remains limited. Future studies are needed to explore their contributions to the gut-kidney axis.

**Table 1.** Gut microbiota alteration in diabetic kidney disease (DKD).

Mechanism of Influence	Gut microbiota	Studies	Amount in DKD Patients	Risk of DKD
SCFAs-producing microbiota	*Clostridium, Eubacterium, Roseburia*	[28]	Decrease	Decrease
	*Ruminococcaceae, Butyricicoccus, Lachnospiraceae*	[72]		
	*Clostridium-XVIII, Gemmiger*	[73]		
	*Roseburia, Faecalibacterium, Blautia, Subdoligranulum*	[74]		
	*Roseburia, Clostridium, Eubacterium, Fusobacterium*	[28]		
	*Coprococcus, Lachnospira*	[75]		
Other potential probiotics	*Pyramidobacter, Prevotellaceae_UCG-001*	[76]	Decrease	Decrease
	*Lachnospira, Romboutsia, Intestinibacter*	[28]		
LPS-producing microbiota	*Proteobacteria, Verrucomicrobia, Fusobacteria*	[29]	Increase	Increase
	*Proteobacteria*	[75,76]		
	*Flavonifractor*	[73]		
Other Potential pathogenic microbiota	*Bacteroides*	[28]	Increase	Increase
	*Eisenbergiella, Christensenella, Clostridium-XIVa, Clostridium-XVIII*	[73]		
	*Alistipes, Bacteroides, Subdoligranulum, Lachnoclostridium, Ruminococcus*	[77]		

The complex relationship between gut microbiota and DKD suggests that modulating gut microbiota composition could serve as a new strategy for treating DKD. By increasing beneficial bacteria or reducing pathogenic bacteria, inflammation and metabolic disturbances that promote DKD progression can be alleviated. This approach holds promise for developing innovative treatments to restore gut health and protect kidney function in diabetic patients.

In addition to the above mechanisms, it is worth noting that DKD progression mirrors the clinical staging of CKD, including declining estimated glomerular filtration rate (eGFR)[78]. Therefore, studies on gut microbiota changes across CKD stages may offer valuable insights into how dysbiosis evolves over the course of DKD. Increasing evidence suggests that the composition and function of the gut microbiota change as CKD progresses. Patients with more advanced stages of CKD, particularly those with significantly reduced eGFR, tend to exhibit lower microbial diversity, higher abundance of urease- and indole-producing bacteria, and a depletion of SCFA-producing species such as *Faecalibacterium prausnitzii* and *Roseburia* [79,80]. This shift toward a more dysbiotic microbiota is associated with increased production and systemic absorption of uremic toxins, including IS and PCS, which can accelerate renal inflammation and fibrosis. Conversely, the uremic environment—characterized by metabolic acidosis, fluid overload, and dietary protein restrictions—may further impair the gut barrier and contribute to microbiota alterations [81]. Importantly, a recent study by Zhang et al. [82] directly compared gut microbiota composition in patients with early- and late-stage DKD. The results demonstrated a progressive loss of microbial diversity and enrichment of pro-inflammatory taxa in advanced DKD, supporting the notion that gut dysbiosis intensifies with disease severity. This study provides direct evidence that gut microbiota alterations are not only a consequence but may also contribute to the progression of DKD. These findings suggest a bidirectional relationship: as kidney function declines, gut dysbiosis worsens, and in turn, dysbiosis exacerbates renal injury. Understanding these stage-specific microbial shifts may offer new opportunities for microbiota-based interventions at different phases of DKD.

## 3. The diagnostic and prognostic utility of the gut microbiome composition for patients with diabetic kidney disease

Researchers are actively investigating novel biomarkers to improve the identification of high-risk individuals and facilitate early detection of DKD. These innovative biomarkers could enhance the evaluation of DKD risk and shed light on the intricate biology underlying the condition, potentially uncovering new therapeutic targets. Patients with DKD experience notable changes in their gut microbiota composition. These alterations could potentially be used as indicators to assist in clinical diagnosis or to validate the condition through biopsy. For those who cannot undergo renal biopsy due to medical contraindications, analyzing the gut microbiota may offer a vital alternative method for assessing the disease [83].

A research conducted in Sichuan, China, involving 14 subjects with biopsy-confirmed DKD, revealed that the *Prevotella-9* could effectively differentiate between diabetes patients and healthy individuals. Furthermore, the study found that the *Escherichia*–*Shigella* (a taxonomic grouping of closely related *Escherichia* and *Shigella* species) and *Prevotella-9* were capable of distinguishing biopsy-confirmed DKD patients from those with diabetes, which aided in the diagnosis of DKD [75]. On the other hand, a study conducted by Lu et al. in Shanxi, China, yielded different results. Their research, which involved 35 cases of biopsy-confirmed DKD, found that the gut microbiota genera *Flavonifracto*r or *Eisenbergiella* could effectively differentiate between patients with DKD and those with diabetes only [73]. The observed disparities may be explained by differences in geographical and nutritional factors. Moreover, three bacterial species: *Clostridium sp. CAG-768*, *Bacteroides propionicifaciens*, and *Clostridium sp. CAG-715* were identified as effective markers for differentiating between patients with DKD and healthy controls. A multiple linear regression study revealed that the joint detection of *Fusobacterium varium, Pseudomonadales*, and *Prevotella sp. MSX73* successfully distinguished T2DM from DKD. Moreover, these bacterial indicators demonstrated a higher area under the receiver operating characteristic curve in differentiating T2DM and DKD compared to the urinary albumin-to-creatinine ratio, albumin, and urinary creatinine ratio [28]. A predictive model using random forest techniques, constructed from the 25 microbial genera with the least correlation, exhibited exceptional accuracy in identifying DKD. The model achieved an area under the receiver operating characteristic curve of 0.972, suggesting that gut microbiome composition could serve as a valuable diagnostic indicator for this condition [74]. These results point to the potential of the gut microbiome as a target for developing new diagnostic methods. However, research has demonstrated that gut microbial biomarkers linked to DKD differ across geographic regions and populations. As a result, additional clinical studies are required to comprehensively assess the diagnostic efficacy of the gut microbiome in relation to DKD. Further research is needed to validate specific microbial signatures as reliable diagnostic markers and to establish standardized microbiota-based therapies for clinical use.

Furthermore, the progression of DKD does not always follow the traditional pattern from microalbuminuria to macroalbuminuria and subsequent renal function decline. Some patients may exhibit atypical progression patterns, such as nonproteinuric DKD or early renal function decline with minimal proteinuria [84]. Current biomarkers, predominantly focusing on albuminuria and renal function indices, may not fully capture these atypical trajectories. Therefore, gut microbiota composition analysis may serve as an important complementary biomarker, helping to more accurately identify and monitor these atypical progression patterns. Future research should further validate specific microbiota signatures that correlate with these non-classical DKD phenotypes.

## 4. Therapeutic approaches involving gut microbiota

Traditional treatment strategies for DKD focus primarily on managing hyperglycemia and hypertension to slow disease progression. Insulin and oral hypoglycemic agents, such as metformin and sulfonylureas, are frequently used to control blood glucose levels, which is a fundamental aspect of DKD management [85]. Controlling blood glucose levels helps slow the progression of DKD [86]. Angiotensin-converting enzyme inhibitors and angiotensin II receptor blocker help manage hypertension, reduce proteinuria, and protect kidney function [85,87,88]. Additionally, lifestyle modifications, including dietary adjustments (e.g. low-sodium, low-protein diets), regular physical activity, and cessation of smoking and alcohol consumption, are crucial [89]. However, these conventional approaches are not always sufficient, especially in the later stages of DKD when patients may require renal dialys [90] is or kidney transplantation [91].

In recent years, growing evidence has highlighted the gut microbiota as a key modulator of DKD progression, paving the way for novel microbiota-targeted therapeutic strategies. Gut dysbiosis, characterized by reduced beneficial bacteria and increased pathogenic species, is strongly linked to metabolic disturbances and chronic inflammation in DKD patients. Thus, therapeutic interventions aimed at restoring a healthy gut microbiota balance have emerged as promising approaches [92].

### 4.1. Probiotics

Research has shown that probiotics can enhance human well-being through several mechanisms [90]. These include alleviating intestinal inflammation, maintaining balance in gut microbiota, facilitating cell repair, and influencing immune responses [93]. These actions play a vital role in both treating and preventing various health conditions. Studies have shown that consuming probiotics can alleviate symptoms through multiple pathways [94]. A controlled, double-blind study with random assignment revealed that probiotics boost the production of  in the gut while decreasing the generation of hydrogen peroxide free radicals, thereby reducing kidney inflammation and scarring [95]. In diabetic rats induced by streptozotocin, the probiotic strain *Lactobacillus reuteri GMNL-263* was observed to decrease hemoglobin A1c and blood sugar levels, as well as inhibit kidney fibrosis caused by elevated blood glucose [96]. A clinical study involving patients with DKD demonstrated that those who ingested soy milk enriched with *Lactobacillus plantarum A7* for 8 weeks exhibited significantly lower levels of cystatin C and the inflammatory adipokine progranulin compared to the control group [97]. Additionally, supplementation with *Lactobacillus casei* has been shown to improve SCFAs and nicotinamide metabolism, reduce kidney damage, and slow the progression of declining renal function [98]. Recently developed compound probiotics have demonstrated potential as complementary treatments to metformin by enhancing butyrate production and improving glucose metabolism in patients. In a mouse model of CKD induced by high blood sugar, probiotic supplementation was found to decelerate the deterioration of kidney function. Taken together, these results indicate that probiotic supplementation may serve as a promising therapeutic strategy for addressing kidney disease stemming from diabetes-related metabolic disruptions.

### 4.2. Prebiotics and postbiotics

Prebiotics are nondigestible food components that promote the growth of beneficial bacteria in the gut [99]. In the context of DKD, prebiotics have shown promise as a potential therapy. They work by selectively nourishing beneficial gut bacteria, which can help restore a healthy gut microbiota balance [100]. This balance is often disrupted in DKD, where gut dysbiosis is linked to inflammation and other complications. Prebiotics, such as inulin-type fructans and resistant starch, have been shown to improve kidney function, reduce inflammation, and decrease levels of uremic toxins in the body [101]. Prebiotics can also increase the production of short-chain fatty acids like butyrate, which have anti-inflammatory properties and can improve gut barrier function [102]. They achieve this by increasing the production of SCFAs, which have anti-inflammatory and gut-protective effects. While research is ongoing, studies suggest that prebiotics like fructooligosaccharides may offer benefits in managing DKD by improving gut health and potentially slowing disease progression [103]. However, more research is needed to confirm these findings and establish optimal prebiotic types and dosages for DKD patients.

Postbiotics are metabolites or components of microorganisms, such as enzymes, peptides, and SCFAs, that can offer health benefits. Unlike probiotics, which are live microorganisms, postbiotics are non-living and exert their effects by interacting with the host’s gut microbiota and immune system [104]. In the context of DKD, postbiotics have shown promise in preclinical studies by demonstrating their ability to improve gut microbiota composition, reduce inflammation, and alleviate kidney damage [105]. Another studies have shown that certain postbiotics can increase the production of short-chain fatty acids like butyrate, which has anti-inflammatory and renoprotective effects [106]. However, further research, particularly in human clinical trials, is needed to fully elucidate the therapeutic potential of postbiotics for DKD and establish appropriate dosage regimens.

### 4.3. Synbiotics

In addition to prebiotics and probiotics, there are other gut microbiota-based therapeutic approaches. Synbiotics connotes to a combination of probiotics and prebiotics, synergistically enhance the colonization and activity of beneficial bacteria, further restoring gut microbiota homeostasis [107]. Studies indicate that the combination of probiotics and prebiotics can work synergistically, leading to greater effectiveness compared to using either probiotics or prebiotics individually [108]. The study by Yang et al. confirmed that treatment with synbiotics containing *Bifidobacterium, Lactobacillus, Streptococcus*, and inulin restored gut microbiota diversity in rats with CKD [109]. The synbiotics exerted renal protective effects by reducing gut indole load and correcting dysbiosis.

### 4.4. FMT

Fecal microbiota transplantation (FMT) is a novel therapeutic approach being investigated for its potential in treating DKD [110]. This procedure involves transferring fecal matter from a healthy donor into the gastrointestinal tract of a recipient with DKD [111]. The goal is to reconstruct a healthy gut microbiota in the recipient, as alterations in the gut microbiota have been implicated in DKD pathogenesis. Studies suggest that FMT can lead to improvements in DKD by reducing glomerulosclerosis and fibrosis, decreasing levels of uremic toxins produced by gut microbes, enhancing cholesterol regulation, and improving cholesterol homeostasis [30]. While the exact mechanisms underlying FMT’s benefits in DKD are still being investigated, studies suggest that it may modulate immune responses, reduce inflammation, and improve metabolic profiles [112]. An additional study demonstrated that FMT ameliorated glomerular injury in STZ-induced diabetic rats [113]. In T2DM mouse model, FMT resulted in decreased blood glucose levels, enhanced glucose tolerance and insulin sensitivity, and mitigated pancreatic islet damage [114]. Despite its potential, it’s important to note that research on FMT for DKD is still in its early stages, and more extensive clinical trials are necessary to confirm its long-term efficacy and safety in humans.

### 4.5. Dietary interventions

Dietary interventions are paramount in the management of DKD. A tailored diet serves as a cornerstone of therapy, alongside glucose control and blood pressure management. A recent study suggests that dietary interventions, such as low-carbohydrate diets, calorie restriction, and intermittent fasting, have the potential to improve DKD [115]. Carbohydrate management, emphasizing complex carbohydrates from whole grains, fruits, and vegetables over refined carbohydrates and sugary foods, is crucial for blood sugar control [116]. Calorie control for weight loss, particularly for overweight or obese individuals, as even a modest reduction can significantly benefit kidney function [117]. Protein intake should be monitored and potentially moderated to reduce the burden on the kidneys [118]. Limiting sodium intake is essential for blood pressure control, while potassium and phosphorus intake may require adjustments depending on the stage of DKD and the individual’s kidney function [119]. Dietary approaches like the Mediterranean diet and the DASH diet, rich in fruits, vegetables, and lean protein sources, have shown benefits for DKD patients [120]. Additionally, there are emerging evidences that foods rich in live microbes, such as fermented foods, has the potential to positively influence the incidence and progression of DKD. A study by Liu et al. found that the consumption of fermented and germinated foxtail millet whole grain can reduce kidney damage in diabetic mice, while also lowering the relative abundance of DKD-associated bacteria [121]. He et al. demonstrated that the fermented soybean product could significantly inhibit kidney inflammation in mice, with a notable reduction in the associated microbial community [122]. These findings highlight the importance of dietary interventions in managing DKD, especially through modulating the gut microbiota. However, further clinical trials are needed to validate the long-term efficacy of these dietary strategies and determine the optimal interventions for different stages of DKD.

### 4.6. Herbal medicine

Herbal medicine, particularly traditional Chinese medicine, has garnered significant attention as a potential adjunctive therapy for DKD. Beyond their conventional applications, numerous herbal remedies have demonstrated the capacity to modulate the gut microbiota, a crucial factor in the pathogenesis of DKD.

Multiple research studies have investigated the effectiveness of different herbal remedies and their active components in alleviating DKD, mainly through their impact on the composition and functionality of gut microbiota [123]. As an example, research has demonstrated that Rheum palmatum L. enhances the integrity of the intestinal barrier, lessens renal fibrosis and inflammation, and reduces the prevalence of detrimental bacteria such as *Akkermansia, Methanosphaera,* and *Clostridiaceae* in experimental CKD models [124].

Additionally, various traditional Chinese herbal preparations, such as QiDiTangShen granules, Qing-Re-Xiao-Zheng formula, Tangshen formula, and Zicuiyin decoction have shown effectiveness in managing DKD by addressing gut microbiota imbalance [124–126]. These herbal formulations typically function by decreasing the population of harmful bacteria while simultaneously encouraging the proliferation of beneficial microorganisms, thereby enhancing renal function and overall metabolic well-being. Specifically, administration of Qing-Re-Xiao-Zheng formula increased beneficial bacteria such as *Akkermansia* and *Rikenellaceae*, which are associated with enhanced gut barrier function and anti-inflammatory effects; concurrently, it decreased harmful bacteria like *Desulfovibrio*, known to produce pro-inflammatory substances [125]. These changes contribute to improved renal function and metabolic health in DKD animal models. On the other hand, Tangshen formula treatment resulted in an increased abundance of beneficial *Bifidobacterium* species and a reduction in harmful *Proteobacteria* [126]. This modulation led to decreased levels of gut-derived toxins, reduced systemic inflammation, and ameliorated renal injury in DN rat models.

In addition to whole plant extracts, individual bioactive components extracted from these plants have demonstrated potential in treating DKD. For instance, resveratrol, a naturally occurring polyphenol found in grapes and red wine, has been shown to enhance kidney function, mitigate glomerular damage, and reduce inflammation in diabetic mouse models. These positive outcomes are partly attributed to resveratrol’s capacity to increase the population of beneficial gut microorganisms such as *Bacteroides, Alistipe*s, and *Parabacteroides*, which exhibit anti-inflammatory properties.

Research has shown that curcumin, an active ingredient found in turmeric, may be beneficial in managing DKD. Clinical studies have revealed that curcumin administration can reduce the excretion of urinary microalbumin and lower plasma lipopolysaccharide concentrations in individuals with T2DM [127]. This beneficial effect is attributed to curcumin’s capacity to influence the gut microbiome by enhancing the population of *Bacteroides, Bifidobacterium,* and *Lactobacillus* [128]. These bacterial species play a role in repairing the epithelial barrier and suppressing inflammatory responses triggered by LPS.

Beyond resveratrol and curcumin, other naturally occurring polysaccharides, including those derived from Moutan Cortex [129] and Bupleurum [130], have demonstrated potential in alleviating DKD through their influence on gut microbiota composition. These observations underscore the promising role of herbal medicines and their active components as a supplementary strategy for DKD management, specifically by targeting the gut microbiome. Nevertheless, additional research, particularly extensive clinical trials, is essential to validate these results and determine appropriate dosing and treatment protocols.

### 4.7. Engineering bacteria and phages

Engineering bacteria and phages have emerged as promising new therapies for treating metabolic disorders like DKD by targeting the gut microbiome. Engineered *Lactobacillus gasseri* can increase insulin levels and improve glucose tolerance in diabetic rats by secreting GLP-1 and reprogramming intestinal cells to become insulin-secretin [131]. Additionally, engineered bacteria encapsulated in microcapsules can stably release therapeutic proteins and lower blood glucose in diabetic rats, demonstrating the potential of engineered bacteria for DKD treatment [132]. Phages are viruses that infect bacteria, can also play a therapeutic role. Transferring cecal viral communities from lean to obese mice reduces weight gain and normalizes glycemic parameters, likely due to phage-bacteria antagonism [133]. Phages can also promote beneficial bacterial biofilm formation [134].

### 4.8. Novel drug approaches

The landscape of DKD treatment is rapidly evolving, with promising research avenues pointing toward novel therapies that target the intricate pathogenesis of the disease. While current management strategies primarily focus on blood glucose control and renin-angiotensin-aldosterone system blockade, future therapies aim to address the underlying molecular mechanisms driving DKD progression. GLP-1 receptor agonists are known for their glucose-lowering effects. However, they also play a significant role in protecting against DKD [135]. Studies show that GLP-1 receptor agonists can improve kidney function and reduce albuminuria in individuals with DKD [136]. These beneficial effects are attributed to their ability to reduce inflammation, oxidative stress, and fibrosis in the kidneys. Clinical results showed that liraglutide could improve DKD symptoms and reduce the incidence of nephropathy and persistent macroalbuminuria in T2DM patients [137]. Furthermore, liraglutide treatment seemed to reverse microbiota dysbiosis in mice with disease states, with decreased *Actinobacteria* and *Desulfovibrio* and increased *S24-7* [138]. It is worth mentioning that liraglutide also increases the relative abundance of *Akkermansia* in db/db mice, a new type of probiotic in the gut, suggesting the possibility of microbiota-modulating effects of liraglutide in the treatment of DKD [139]. Another approach involves targeting epigenetic modifications, particularly histone modifications, which play a crucial role in regulating gene expression and cellular function [140]. Another strategy focuses on mitigating hypoxia, a hallmark of DKD, using hypoxia-inducible factor prolyl hydroxylase inhibitors to enhance renal oxygenation and potentially improve renal function [141]. Additionally, activating the Nrf2 pathway, a master regulator of antioxidant defenses, through Nrf2 activators, has shown promise in preclinical and clinical studies [142]. Beyond these molecular targets, regenerative medicine approaches, such as renal cellular therapy utilizing mesenchymal stem cells, offer the potential to repair and restore damaged kidney structures, potentially reversing DKD progression. As our understanding of DKD pathogenesis deepens, the development of personalized medicine approaches, tailored to an individual’s genetic and molecular profile, holds immense potential for optimizing treatment strategies and improving patient outcomes.

### 4.9. Clinical implications and practical recommendations for physicians

Gut microbiota-targeted strategies may offer promising adjunctive approaches for DKD management. From a clinical standpoint, gut dysbiosis has been associated with both metabolic dysregulation and systemic inflammation, two key drivers of DKD progression. Notably, many patients with advanced DKD present with gastrointestinal symptoms such as constipation, bloating, or diarrhea, which may reflect underlying microbial imbalances [143]. These observations have led to increased attention on the gut as a potential site for therapeutic intervention. Several clinical trials have demonstrated that interventions such as probiotics, prebiotics, and synbiotics can reduce markers of inflammation, improve gut barrier function, and potentially delay CKD progression [144]. In Chinese clinical practice, certain traditional Chinese medicines, such as Niaoduqing granules, have been used to regulate intestinal microbiota, reduce uremic toxin levels, and improve renal outcomes [145]. While these formulations are not yet widely adopted in Western nephrology, preliminary studies support their role in modulating microbial metabolism and attenuating kidney inflammation.

Physicians should be encouraged to consider the gut as a potential therapeutic target, particularly in patients with digestive symptoms, recurrent inflammation, or poor metabolic control. Beyond the conventional management of blood glucose, blood pressure, lipids, and the use of RAS inhibitors, incorporating gut microbiota testing into routine diagnosis and treatment monitoring for DKD could provide valuable insights for personalized treatment. For patients with abnormal gut microbiota profiles, targeted interventions, such as probiotics, prebiotics, or dietary modifications, could be applied to restore a healthy microbiota balance. However, the feasibility and cost-effectiveness of routine microbiota testing need further evaluation, and standardized guidelines should be developed to ensure accurate interpretation of test results.

Table 2 summarizes potential clinical considerations for physicians managing DKD patients with suspected gut dysbiosis. Note: These suggestions are exploratory and should be interpreted in the context of patient-specific factors and regional clinical practices**.**

**Table 2.** Suggested clinical considerations for managing gut dysbiosis in DKD.

Clinical Situation	Suggested Action
DKD with constipation, bloating, or diarrhea	Consider evaluating for gut dysbiosis; dietary adjustment or probiotic support may help
Elevated uremic toxin levels (IS, PCS)	Supportive strategies may include fiber intake, prebiotics, or gut-targeted approaches
DKD with persistent inflammation or poor glucose control	Consider SCFA-enhancing interventions, such as synbiotics or dietary fiber
Advanced DKD with malnutrition or low diversity	Microbiota-focused therapies (e.g. TCM like Niaoduqing granules) may be considered
Individualized treatment planning	Monitor gut-related symptoms and tailor interventions to patient-specific needs

## 5. Challenges and opportunities

### 5.1. Existing challenges and limitations

Despite the significant potential of microbiota-targeted therapies in the treatment of DKD, numerous obstacles and challenges remain. First, the high heterogeneity of individual gut microbiota composition complicates the prediction of treatment outcomes. Baseline microbiota composition varies significantly among patients due to factors such as genetics, diet, and lifestyle, leading to variability in therapeutic efficacy [146–148]. The use of medications such as proton pump inhibitors, metformin, laxatives, and antineoplastic drugs, in addition to antibiotics, can also significantly impact the gut microbiota [149]. Thus, it is an important consideration whether treatment goals and microbiota-targeted therapies should be adjusted for different patient subgroups. However, current research has not yet explored this level of refinement due to the limited number of subgroup-specific studies.

Second, long-term adherence to microbiota-targeted therapies, such as probiotics and prebiotics, is essential for sustained effects [150]. However, patients may face challenges in maintaining consistent treatment due to lifestyle, financial constraints, and the complexity of the regimen. Additionally, although probiotics have demonstrated positive outcomes in preclinical studies, their ability to establish long-term colonization in DKD patients remains uncertain, particularly in immunocompromised individuals, where probiotics may pose a potential infection risk [151].

One of the key limitations in gut microbiota studies is the lack of standardized methodologies across different research efforts. To enhance the comparability and reliability of results, it is crucial to develop and adopt standardized protocols for sample collection, processing, and analysis. While 16S rRNA gene sequencing is widely used due to its cost-effectiveness, it provides limited insight into microbial function [152]. Metagenomics, on the other hand, offers higher taxonomic and functional resolution but generates large, complex datasets that require advanced bioinformatics for analysis. The lack of standardized methodologies across studies complicates cross-study comparisons, making it difficult to draw consistent conclusions [153]. Future efforts should focus on harmonizing these methodological approaches to improve the reliability and reproducibility of microbiota-related studies.

Moreover, establishing causality between gut microbiota changes and DKD progression remains a key challenge. Although studies have identified associations between specific microbial taxa and DKD, however, most studies currently are observational, making it difficult to infer causal relationships. Future studies should consider using methods such as Mendelian randomization, which can help infer the role of microbiota in DKD progression by utilizing genetic variations as instrumental variables [154,155]. Additionally, longitudinal intervention studies will also be helpful in elucidating how specific microbial populations influence the occurrence and development of DKD.

### 5.2. Future directions and research opportunities

While challenges persist, microbiota-targeted therapies for DKD have a hopeful future ahead. First, given the high heterogeneity of gut microbiota, personalized medicine holds significant potential in the treatment of DKD. Future research should focus on exploring whether microbiota-targeted therapies should be refined based on patient subgroups, including those of different ages, disease durations, and comorbidities, to enable more precise treatment strategies. Advancements in high-throughput sequencing and big data analysis will further allow individualized treatment plans based on specific microbial biomarkers, optimizing efficacy while minimizing side effects [156,157].

Secondly, while promising, the long-term efficacy and safety of novel microbiota-targeted therapies, such as probiotics, prebiotics, synbiotics, and FMT, require further investigation. For instance, probiotics, though generally considered safe, may pose risks for immunocompromised individuals, such as unwanted colonization or infections [151]. Long-term follow-up studies are needed to evaluate the safety of these therapies in vulnerable populations. Similarly, FMT has shown positive short-term outcomes in restoring microbiota balance, but its long-term effects on microbiota stability and recipient health remain unclear. Research should focus on assessing the sustainability of microbiota restoration and potential risks, such as microbial shifts or adverse events in the long term [112]. Future studies should prioritize long-term safety assessments and consider the risks of microbiota interventions, particularly for patients with comorbidities or compromised immune systems.

Moreover, future research should expand beyond the gut microbiota to explore other microbial communities, such as those in the oral cavity, skin, and respiratory tract [158], which may interact with the gut microbiota and potentially influence inflammatory pathways, immune responses, and metabolic processes that impact DKD. Understanding the interactions between different microbiomes and their collective impact on DKD could provide novel therapeutic targets and strategies for treatment. Additionally, integrating microbiota analysis into clinical practice through standardized testing methods could facilitate personalized treatment development.

Furthermore, artificial intelligence (AI) offers novel approaches for managing gut microbiota in DKD patients by facilitating microbiome analysis, biomarker identification, and predictive modeling [159]. Machine learning algorithms can uncover microbial signatures associated with DKD, enabling early diagnosis and personalized interventions. Although biomarkers such as circulating tumor necrosis factor receptors (TNFR1, TNFR2) and kidney injury molecule-1 (KIM-1) provide predictive value, their accuracy remains limited. Integrating these established biomarkers with additional clinical parameters and microbiota-related indicators using AI could significantly enhance predictive accuracy and improve monitoring of DKD progression. As research advances, AI integration may further promote precision medicine in microbiome-based DKD management.

Novel therapeutic agents such as SGLT2 inhibitors, non-steroidal mineralocorticoid receptor antagonists, and GLP-1 receptor agonists have demonstrated significant renal protective effects and are increasingly utilized in clinical practice [160–162]. However, important clinical questions remain regarding their optimal timing, combination strategies, dosage adjustments, and long-term safety across varying eGFR levels and degrees of proteinuria. Notably, current research on the impact of these medications on the gut microbiota composition and function remains limited. Given the crucial role of gut microbiota in DKD progression and treatment response, future studies should investigate how these novel pharmacological treatments influence gut microbiota dynamics and whether microbiota changes may mediate or modify their therapeutic efficacy and safety profiles.

Additionally, future microbiota-targeted clinical trials should carefully consider the heterogeneity of DKD patients and clinical complexity by increasing sample sizes, enrolling diverse patient populations, and performing longitudinal monitoring of gut microbiota changes in patients with rapid disease progression. This approach could accelerate translation from clinical trials into clinical practice.

In summary, while microbiota-targeted therapies face numerous challenges, their potential in the treatment of DKD is highly encouraging. With advancements in research methodologies, clinical trials, and microbiota analysis techniques, personalized therapeutic strategies may slow DKD progression and significantly improve patient outcomes.

## 6. Conclusion

Gut microbiota plays a critical role in DKD by influencing systemic inflammation, insulin resistance, and kidney damage through mechanisms such as gut permeability and microbial metabolites like SCFAs and LPS. Specifically, bacterial taxa like *Alistipes* and *Subdoligranulum* are involved in key processes, including oxidative stress, inflammation, and cytokine signaling, which exacerbate DKD progression. Targeting these pathways through probiotics, prebiotics, synbiotics, and FMT shows potential for slowing DKD progression. While existing studies suggest the potential of microbiota-based therapies in improving gut barrier integrity, reducing endotoxemia, and enhancing kidney function, further clinical research is needed to confirm these findings and assess their long-term efficacy. The individual variability in gut microbiota composition presents challenges for the widespread application of such therapies. Personalized treatment strategies and large-scale clinical trials are essential for optimizing the use of microbiota-targeted interventions. Future research should also explore the gut-kidney axis in more detail, focusing on the mechanistic roles of specific microbial species in DKD to develop more effective therapeutic strategies. Additionally, AI can aid in microbiota analysis and personalized interventions, enhancing precision in DKD management.

## Data Availability

This review article is based on publicly available data from various studies and resources in the field of DKD and gut microbiota. No new primary data were generated during the course of this review. All referenced data are sourced from public repositories. For more details or specific queries regarding the data, please contact the corresponding author *via* email.
